# Editorial: Recent developments in pancreatic cancer radiotherapy

**DOI:** 10.3389/fonc.2023.1160808

**Published:** 2023-03-09

**Authors:** James C. L. Chow, Antonio Pontoriero

**Affiliations:** ^1^ Radiation Medicine Program, Princess Margaret Cancer Centre, University Health Network, ON, Toronto, Canada; ^2^ Department of Radiation Oncology , University of Toronto, ON, Toronto, Canada; ^3^ Radiation Oncology Unit, Department of Biomedical, Dental and Morphological and Functional Imaging Sciences, University of Messina, Messina, Italy

**Keywords:** pancreatic cancer, radiotherapy, treatment outcome and efficacy, image guidance, retrospective study, stereotactic body radiation therapy, carbon ion therapy, image registration

This book explores the recent advancements in pancreatic cancer radiotherapy, presenting innovative treatment techniques and clinical outcome assessments. Pancreatic cancer is difficult to detect in its early stages as the cancer is usually asymptomatic until it spreads in the patient. So far, no screening tests exist which can detect pancreatic cancer early when it is most curable. Surgery, on its own, is rarely curative in pancreas cancer both due to the anatomic location of the pancreas (at the posterior of the abdomen behind the stomach), and as the disease is generally metastatic at diagnosis. Typically, pancreatic cancer is treated with a combination of surgery, chemotherapy and radiotherapy. Radiotherapy is the most utilized cancer treatment option as over 50% of all cancer patients will receive radiotherapy during their treatment course. With the recent advances of radiation delivery, internal organ motion monitoring, computer calculation, medical image processing, treatment strategy, and treatment planning, encouraging progresses have been made in the pancreatic cancer radiotherapy.

For patients with unresectable pancreatic cancer undergoing definitive radiotherapy, Chen et al. carried out a detailed risk analysis on the treatment outcomes based on potential factors such as overall survival, local progression, distant metastases, carbohydrate antigen, neutrophil-to-lymphocyte ratio and others. They analyzed the biological effective doses in this retrospective study, and found that incorporating new systemic treatments during and after higher biological effective doses from radiotherapy for locally advanced pancreatic cancer is warranted. For novel treatment technique in pancreatic cancer, Feng et al. developed a Haar feature-based method to track the endoscopic ultrasound probe in diagnostic CT and MRI scans for guiding the hydrogel injection in pancreatic cancer radiotherapy. The advantage of this method is that no external tracking hardware is needed when tracking the ultrasound probe. They tested and implemented this method using phantom and patient images, and concluded that their method can find the best matched endoscopic ultrasound image from the dictionary based on simulated images. The pancreas movement was tracked and studied using the synchrony respiratory tracking system in CyberKnife treatment by Jing et al. The pancreatic displacement was calculated from the patients’ x-ray image set and the mean motion amplitudes in the SI, LR and AP directions were determined. They found that the tracking accuracy was affected by the tumour motion amplitude, location and treatment time. Jing et al. therefore concluded that tumours at different locations should be treated differently for the best dose coverage. A retrospective study on stereotactic body radiotherapy combined with chemotherapy was carried out by Lee et al. The overall survival and local progression-free survival were determined for patients with the median follow-up period of 21.1 months. Lee et al. found that stereotactic body radiotherapy followed by chemotherapy was an effective treatment strategy for selected patients with unresectable pancreatic cancer. Moreover, they found that simultaneous integrated protection and the MR-guided adaptive technique were worthwhile to implement in order to minimize the risk of adverse events. Carbon-ion beam has the advantage of a sharp dose distribution due to the characteristic of Bragg peak in pancreatic cancer radiotherapy. In the treatment planning of carbon-ion therapy, Kusano et al. used the CT value replacement method to improve the plan dosimetry due to the influence of the gastrointestinal gas in the patient. They concluded that their method can directly be implemented in clinical practice without additional software and equipment. A retrospective patient study was carried out by Broggi et al. to evaluate patient outcomes for locally advanced pancreatic cancer treated with a combination of chemotherapy and hypofractionated radiotherapy in 15 fractions. Based on the dose-volume results, Broggi et al. found that the risk of duodenal or gastric toxicities was related to the duodenum or stomach dose-volume histogram. The radiation delivery was evaluated by Arumugam et al. using an in-house position monitoring system in pancreas stereotactic body radiotherapy. This in-house system is an online image-based position monitoring system using the radiopaque marker and the Elekta XVI imaging system. In the evaluation, the dosimetric impact due to position deviations and actual delivered dose after position corrections were assessed. From the dosimetric results, Arumugam et al. concluded that their position monitoring system can improve the treatment accuracy using only a general linear accelerator. Another retrospective study was carried out by Cao et al. to investigate the dose in the organs-at-risk and gastrointestinal toxicity, when re-irradiation of stereotactic body radiotherapy was needed for pancreatic cancer patient usually having local recurrence. Using the deformable image registration method, Cao et al. determined various dose-volume variables such as V_10_ of the stomach and D_mean_ of the intestine in the re-irradiation. Jung et al. conducted a clinical outcome evaluation for FOLFIRINOX as a popular systemic regimen followed by stereotactic body radiotherapy in locally advanced pancreatic cancer. In this retrospective study, patient outcome parameters such as overall survival, progression-free survival, resection rate, stereotactic body radiotherapy-related adverse events and prognostic factors were determined. From the result analysis, Jung et al. concluded that the induction of FOLFIRINOX followed by stereotactic body radiotherapy for locally advanced pancreatic cancer can lead to a better survival rate with manageable toxicities. On the radiation delivery, Ma et al. proposed to carry out intensity-modulated electron therapy using mechanical scanning with a robotic arm on a linear accelerator. The beam scan is based on a zigzag pattern generated by an algorithm controlling various delivery parameters such as beam position, beam energy and step-and-shoot discrete scanning. The algorithm was evaluated using CT image set of 10 pancreatic cancer patients and from the result Ma et al. concluded that intensity modulated electron therapy is potentially feasible in pancreatic cancer treatment undergoing intraoperative radiotherapy.

We hope the results and findings in this book can be useful to our colleagues, clinicians and researchers in pancreatic cancer radiotherapy. We would also like to thank and congratulate all authors who contributed their insightful and significant works to this book.

## Author contributions

Both JC and AP contributed to manuscript draft, read, and approved the submitted version.

